# Array-Based Comparative Genomic Hybridization Analysis Reveals Chromosomal Copy Number Aberrations Associated with Clinical Outcome in Canine Diffuse Large B-Cell Lymphoma

**DOI:** 10.1371/journal.pone.0111817

**Published:** 2014-11-05

**Authors:** Arianna Aricò, Serena Ferraresso, Silvia Bresolin, Laura Marconato, Stefano Comazzi, Geertruy Te Kronnie, Luca Aresu

**Affiliations:** 1 Department of Comparative Biomedicine and Food Science, University of Padova, Padova, Italy; 2 Department of Women’s and Children’s Health, University of Padova, Padova, Italy; 3 Centro Oncologico Veterinario, Sasso Marconi, Italy; 4 Department of Animal Pathology, Hygiene and Public Health, University of Milano, Milan, Italy; National Institute of Genomic Medicine, Mexico

## Abstract

Canine Diffuse Large B-cell Lymphoma (cDLBCL) is an aggressive cancer with variable clinical response. Despite recent attempts by gene expression profiling to identify the dog as a potential animal model for human DLBCL, this tumor remains biologically heterogeneous with no prognostic biomarkers to predict prognosis. The aim of this work was to identify copy number aberrations (CNAs) by high-resolution array comparative genomic hybridization (aCGH) in 12 dogs with newly diagnosed DLBCL. In a subset of these dogs, the genetic profiles at the end of therapy and at relapse were also assessed. In primary DLBCLs, 90 different genomic imbalances were counted, consisting of 46 gains and 44 losses. Two gains in chr13 were significantly correlated with clinical stage. In addition, specific regions of gains and losses were significantly associated to duration of remission. In primary DLBCLs, individual variability was found, however 14 recurrent CNAs (>30%) were identified. Losses involving IGK, IGL and IGH were always found, and gains along the length of chr13 and chr31 were often observed (>41%). In these segments, MYC, LDHB, HSF1, KIT and PDGFRα are annotated. At the end of therapy, dogs in remission showed four new CNAs, whereas three new CNAs were observed in dogs at relapse compared with the previous profiles. One ex novo CNA, involving TCR, was present in dogs in remission after therapy, possibly induced by the autologous vaccine. Overall, aCGH identified small CNAs associated with outcome, which, along with future expression studies, may reveal target genes relevant to cDLBCL.

## Introduction

Diffuse Large B-cell Lymphoma (DLBCL) is the most common canine lymphoproliferative tumor, accounting for approximately 50% of non-Hodgkin’s lymphomas occurring in this species. In dogs, DLBCL exhibits a different clinical behavior based on the variable responses to the same treatments, even within the same clinical stage [1]. Recently, gene expression profiling in canine DLBCL has shown two biologically distinct subtypes. The constitutive activation of the nuclear factor kB pathway has also been identified as a distinctive feature, but other possible mechanisms may underlie the pathogenesis of this tumor [2], [3]. Furthermore, dogs with DLBCL have been studied in a therapeutic clinical trial using an autologous vaccine, possibly being relevant to translational therapy [4].

In human patients, molecular heterogeneity within lymphoma histotypes has been ascribed to an array of chromosomal abnormalities, such as chromosomal translocations and deletions of tumor suppressor genes [5]. To date, cytogenetic aberrations in DLBCL have been scarcely investigated in dogs. In 2011, Thomas and colleagues [6] made substantial progresses by analyzing a high number of canine lymphomas with a Bacterial Artificial Chromosome (BAC) based microarray platform for comparative genomic hybridization (CGH). However, more recently, microarray-based formats, using large insert genomic clones, cDNAs or oligonucleotides, have replaced metaphase chromosomes providing advantages, such as a higher resolution, and the ability to directly map the copy number changes to the genome sequence. Through high-resolution genome-wide DNA microarray analyses, many novel tumor-specific microdeletions and amplifications have been discovered in different human tumors [7]–[11]. In human lymphoma, the improved resolution of array CGH (aCGH) formats has increased the number of the identified genomic aberrations and, importantly, a number of copy number alterations (CNAs) detected by aCGH, that were undetectable by metaphase CGH, have been associated with prognosis and predicted outcome [12]. A similar approach has not been considered for canine lymphomas yet.

The first aim of this work was to identify genomic regions or even gene-specific CNAs in canine DLBCL at the currently highest available resolution. To this end, we applied oligonucleotide aCGH (oligo aCGH) to pair primary DLBCLs and relative skin punch biopsies. An association between regions of DNA CNAs and response to therapy was also investigated.

Numerous observations have demonstrated that at different time points during chemotherapy new alterations affecting specific chromosomal regions become evident. In human lymphoma, these alterations represent the outgrowth of more malignant subclones associated with a more aggressive phenotype [13]. In veterinary medicine, so far, no data have documented molecular genetic alterations acquired at relapse. To address also this point, in a reduced number of dogs, we compared genomic imbalances between DLBCLs matched samples at initial presentation and at relapse.

## Materials and Methods

### Dogs and samples

The study cohort consisted of 12 dogs with newly-diagnosed multicentric DLBCL that underwent complete staging work-up, and that were treated with chemotherapy or chemo-immunotherapy. The diagnosis of DLBCL was obtained by histopathological and immunohistochemical analysis (CD20 and CD79) of one enlarged lymph node, that was surgically removed at initial presentation. A portion of the tumor was preserved frozen in RNAlater solution (Life Technologies, Carlsbad, CA) under sterile conditions. Medical records of all dogs were reviewed to obtain relevant clinical information, including breed, sex, age, clinical stage, substage and treatment. Time to progression (TTP) was measured as the interval between initiation of treatment and progressive disease (PD). Dogs not experiencing PD at the end of the study and dogs lost to follow-up before PD were censored for TTP analysis. Lymphoma-specific survival (LSS) was measured as the interval between initiation of treatment and lymphoma-related death. Censoring was done for dogs that were lost to follow-up, for dogs that died from lymphoma-unrelated causes, and for dogs that were still alive at the end of the study. After having completed the treatment protocol and at relapse, dogs underwent complete end-staging or re-staging, respectively, including PCR for antigen receptor rearrangement on peripheral blood, bone marrow and lymph node tissue. At diagnosis, a skin punch biopsy was also obtained from all dogs.

The study was approved by the Ministry of Education, Universities and Research, University Committee, Rome, Italy (protocol 20086MSFH3), and a mandatory written consent from owners was obtained.

### DNA extraction

Genomic DNA was extracted from lymph nodes and skin punches using the DNeasy Blood & Tissue Kit (QIAGEN, Valencia, CA, USA) according to the manufacturer’s instructions. DNA concentration and quality were measured by a Nanodrop ND-1000 spectrophotometer (Nanodrop Technologies, Wilmington, DE), Qubit fluorometer (Life Technologies) and by denaturing gel electrophoresis.

### Array Comparative Genomic Hybridization

In total, the DNA obtained from 19 lymph node specimens, together with the paired skin biopsies were analyzed using a 180,000-feature SurePrint G3 Canine CGH Microarray (4×180 K, Agilent Technologies), comprising repeat-masked 60-mer oligonucleotides distributed at approximately 2.7 kb intervals throughout the dog genome assembly. Tumor and reference DNA samples were labelled independently with cyanine 3-deoxyuridine triphosphate (dUTP) and cyanine 5-dUTP, respectively, using SureTag Complete DNA Labeling Kit. Oligo aCGH analysis was performed following the manufacturer’s recommendations, using an Oligo aCGH/ChIP-on-chip Hybridization Kit (Agilent Technologies), Canine Cot-1 DNA (Applied Genetics Laboratories, Inc., FL, USA) and Oligo aCGH/ChIP-on-chip Wash Buffer 1 and Oligo aCGH/ChIP-on-chip Wash Buffer 2 (Agilent Technologies). Arrays were scanned at 3 µm resolution using an Agilent G2565CA scanner, and image data were processed using Feature Extraction version 10.7, Genomic Workbench version 6.0 and a CGH11050oct2012 protocol (Agilent Technologies). Data were filtered to exclude probes exhibiting non uniform hybridization or signal saturation and were normalized using the centralization algorithm with a threshold of eight. The ADM-1 algorithm was applied to define CNAs using a ‘three probes minimum’ filter and a threshold of eight without a fuzzy zero correction. To minimize the risk of generating data representing naturally occurring copy number variants not associated with the tumor, reference DNA samples used in this study were derived from the dog’s own skin biopsy. Thus, each lymphoma specimen was associated with the related skin biopsy.

### Data Analysis

The Cy5/Cy3 intensity ratios for each spot were converted into log2 ratios. Aberrant chromosome intervals (except for X chromosome) were selected by using Agilent Genomic Workbench v. 7.0. Copy number gain was defined as a log 2 ratio >0.25 and a copy number loss was defined as a log 2 ratio <−0.25. High-level gain or amplification were defined as a log 2 ratio >1 and 1.5 respectively.

Chromosomal locations were defined in terms of their Megabase (Mb) position. For comparison of genomic imbalances shared between human and dog, orthologous regions on human chromosomes were identified using the Ensemble Genome Browser (http://www.ensembl.org/index.html).

In order to find biological ontologies and pathways enriched in recurrent CNAs, functional annotations of all genes mapping to those aberrant regions was performed using the Functional Annotation Tool Database for Annotation, Visualization, and Integrated Discovery (DAVID) Bioinformatics Resources 6.7, NIAID/NIH (http://david.abcc.ncifcrf.gov/summary.jsp), server “Biological process” (BP), “Molecular function” (MF) and “Cellular component” (CC) annotations were performed by setting gene count = 3 and ease = 0.05. The same parameters were employed also for “Kyoto encyclopedia gene and genome” (KEGG) pathway analysis.

### Statistical analysis

For statistical analysis, dogs were divided into two groups based on response to treatment. Dogs that failed to achieve a complete response to treatment or that achieved an initial response but relapsed before treatment completion were defined as “therapy-resistant” (n 6), whereas dogs that maintained a complete response after the end of treatment were defined as “therapy-responsive” (n 6). Associations between specific CNAs and the two groups were tested using Fisher exact test. Analyses were performed using SPSS software (version 21, SPSS, Chicago, IL, USA). To identify genes with frequent abnormalities in multiple samples, minimal common regions (MCRs) were identified. MCRs represented the maximal overlapping zone across samples within an abnormal (gained/lost) chromosomal region [14], and were defined by means of Agilent Genomic Workbench v. 7.0 by applying T-Test Common Aberration algorithm with Overlap threshold of 0.8 and p-value <0.05. In the present study, aberrations shared in at least four cases (≥30% of cases) were considered as recurrent MCRs.

Genomic Identification of Significant Targets in Cancer (GISTIC) algorithm as implemented in CGHtools software was then used to identify CNAs significantly associated with clinical outcome. The GISTIC module identifies regions of the genome that are significantly amplified or deleted across a set of samples. Each aberration is assigned a G-score that considers the amplitude as well as the frequency of its occurrence across samples. False Discovery Rate q-values are then calculated for the aberrant regions, and regions with q-values below a user-defined threshold are considered significant. Log2ratios ≥0.25 and ≤–0.25 were assigned as threshold for gain and loss detection while a False Discovery Rate (FDR) ≤0.05 was set as limit of significance.

## Results

### Clinical results

Twelve dogs with newly-diagnosed, previously untreated, multicentric centroblastic DLBCL were prospectively enrolled. Of these, 7 were from a series that was included in a previous clinical trial [4]. Five dogs that received the same chemo-immunotherapeutic treatment, but that were not included in the published trial, were also enrolled. Overall, 9 dogs received the same chemo-immunotherapy protocol, and 3 were treated by means of chemotherapy only. Chemotherapy did not differ among dogs. The main clinical characteristics of these dogs are listed in Table S1 in File S1.

### Genomic pattern of aberrations in DLBCL

**Figure 1:**
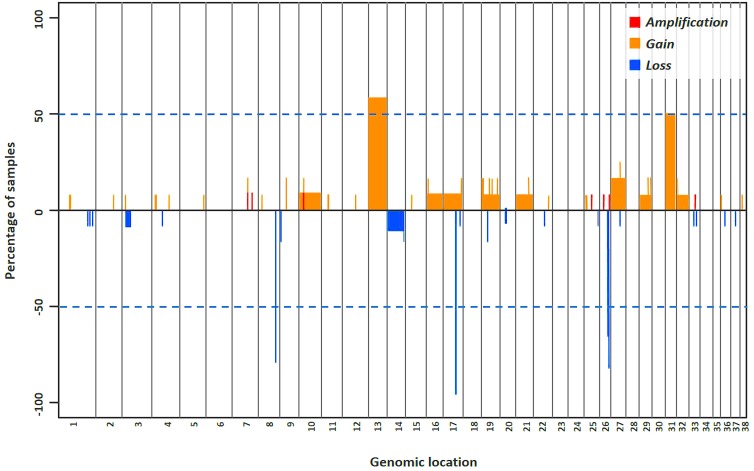
Genomic aberrations in cDLBCL. Frequency plot of CNAs in 12 pre-treatment DLBCLs. Copy number gains/amplifications and losses are indicated as red and blue, respectively. The thresholds of log2 ratio values for single-copy number gains and losses were >0.25 and <−0.25, respectively; the thresholds for high-level gain or amplification were defined as a log 2 ratio >1 and 1.5 respectively.

In pre-treatment DLBCLs, oligo aCGH detected both small and whole-chromosome areas of gains as well as deletions. The pattern of genomic aberrations consisted of 90 different genomic imbalances (mean per tumor, 17; range, 2–29), and included 46 genomic gains (mean per tumor, 9; range, 4–22), and 44 losses (mean per tumor, 7; range 2–18) (Figure 1). The DLBCL in one dog showing the highest number of aberrations relapsed in a short time (31 days) (Table S2 in File S1).

In the analysis of the three matched relapse samples, a lower number of chromosomal changes was detected compared with primary DLBCLs: 15 different genomic imbalances (mean per tumor, 10; range, 7–14), distributed as 8 gains (mean per tumor, 6; range 5–8) and 7 losses (mean per tumor, 4; range 2–6). In all cases, at least one common genomic abnormality was found both in the primary DLBCL and at relapse, confirming the clonal relationship between matched tumors. The most frequent loss at relapse was in Canis Familiaris (CFA) 17q15 (37.6 to 37.8 Mb), whereas the most frequent gain was along the length of the whole chr13. In one dog, between the time of diagnosis and relapse, the acquisition of 3 new genomic alterations was also observed. All were identified as losses (CFA 8q11, 2.4 to 2.9 Mb; CFA 8q33.2, nearly 70.7 Mb; CFA 16q11, nearly 1.9 Mb). One dog displayed the same profile in the pre-treatment DLBCL and at relapse (Table S3 in File S1).

At the end of treatment, four dogs in clinical remission underwent end-staging lymphadenectomy. In all cases, the histological diagnosis was compatible with follicular hyperplasia, and clonality was negative. Analysis of aCGH data detected 10 different genomic imbalances in total (mean per sample, 4; range 4–4), distributed as 1 gain (mean per sample 1) and 9 losses (mean per sample, 3: range 3–4). A total of five new losses were observed in the dogs that were in remission at the end of treatment (CFA 9q11, nearly 1.0 Mb; CFA 16q12, 6.7 to 6.9 Mb; CFA 19q21, 20.2 to 20.3 Mb; CFA 37q14, nearly 18.9 Mb). The genomic loss in CFA 8q11 (2.4 to 2.9 Mb) was the most frequently found (75%). One new gain (CFA 25q24, 51.1 to 51.2 Mb) was found in one dog (Table S3 in File S1).

**Figure 2:**
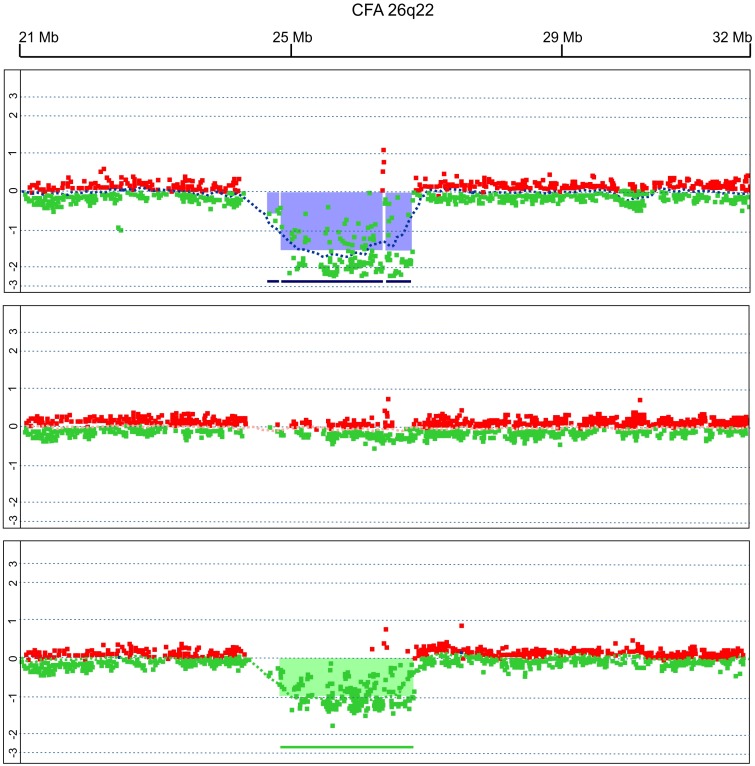
Example of a single genomic aberration (CFA 26q22) at different time points in dog 2. The log2 ratio value is plotted on the y-axis; the x-axis represents the genomic position of probes on the array. The genomic profiles indicate a focal loss in chr26, involving IGL, in pre-treatment DLBCL (upper plot) and during relapse (lower plot); the loss was not detected in clinical remission at the end of therapy (middle of plot).

One example of a single genomic aberration at different time point is shown in Figure 2.

### Minimum common regions (MCRs) analysis

To identify genes with frequent abnormalities in multiple samples, MCRs were identified in pre-treatment DLBCLs. In Table 1, MCRs and the known genes that reside within these DNA segments are shown. Nine MCRs were gained in ≥41% of cases; the three most common regions were located in chr13 (CFA 13q13, 24.3 to 26.2 Mb; CFA 13q13-q21.2, 26.2 to 34.4 Mb; CFA 13q21.1, 37.4 to 38.2 Mb), and were present in ≥66% of cases. The other 4 gains located in the same chromosome (0.03 to 16.6 Mb; CFA 13q12-q13, 16.6 to 24.4 Mb; CFA 13q21.1, 34.4 to 37.4 Mb; CFA 13q21.1-22.2, 38.2 to 63.0 Mb) had a frequency that ranged between 50% and 58%. Conversely, gain in the whole chr31 was less frequently found (41%). Five MCRs were lost in ≥50% of samples. The three most frequently lost MCRs were located in CFA 8q33.3 (72.9 to 74.1 Mb), CFA 17q15 (37.6 to 37.8 Mb) and CFA 26q22 (25.4 to 27.6 Mb), and observed in ≥91% of cases. Immunoglobulin heavy chain (IGH), immunoglobulin kappa (IGK) and lambda (IGL) genes are encoded in these segments.

**Table 1:** List of recurrent MCRs in pre-treatment DLBCLs.

n°aberration*	Chr	Cytogeneticband	Startposition (Mb)	Stopposition (Mb)	Type ofCNA	P-value	Frequency(%)	Genes
22	8	8q33.3	72.95	74.12	Loss	p<0.01	91%	IGHG
35	13	Whole	0.03	16.67	Gain	p<0.001	50%	UBR5, AHGPT1
36	13	13q12-q13	16.68	24.48	Gain	p<0.001	50%	ZHX2
37	13	13q13	24.35	26.26	Gain	p<0.001	75%	MYC
38	13	13q13-q21.1	26.28	34.46	Gain	p<0.001	66%	LDHB, ADCY8, ZFAT
39	13	13q21.1	34.47	37.43	Gain	p<0.001	58%	NAPRT1, BAI1, PTK2
40	13	13q21.1	37.44	38.22	Gain	p<0.001	66%	BOP1, HSF1, FBXL6, FOXH1
41	13	13q21.1-q22.2	38.22	63.08	Gain	p<0.001	58%	AREG, DCK, KDR, KIT, LNX1, PDGFRα, SPINK2, UGT2A1
56	17	17q15	37.53	37.60	Loss	p<0.01	50%	IGKV4-1
57	17	17q15	37.69	37.85	Loss	p<0.001	100%	IGKV6D-41
70	25	25q24	50.32	50.50	Loss	p<0.001	41%	GPC1
71	26	26q22	25.41	27.63	Loss	p<0.001	91%	IGLV11-55
82	31	31q12-q15.2	12.00	35.91	Gain	p<0.01	50%	CHAF1B
84	31	whole	0.09	39.82	Gain	p<0.05	41%	CHAF1B, OLIG2, ERG, RUNX1, CADM2, DNMT3L, DYRK1A

*referred to Table S1 in File S1.

### Genomic pattern of aberration correlated with clinical outcomes

Clustering techniques are commonly used to identify molecular features correlated with clinical outcomes. In the present study, both probes log2ratio and discrete values assigned to genome region were employed to perform unsupervised hierarchical clustering of primary DLBCLs, but both approaches failed to provide consistent results, probably due to the limited number of samples in this study (data not shown).

Microarray data were then interrogated using the GISTIC analysis to identify CNAs whose frequency was significantly increased in the therapy-resistant or the therapy-responsive group. A total of 7 discrete regions whose distribution showed significant variation between the two groups were identified. In particular, the extensive gain of chr13 was significantly more frequent in therapy-responsive DLBCLs (5/6) compared to therapy-resistant DLBCLs (2/6). Conversely, the gain of the entire chr31 was more frequently found in the therapy-resistant group. The extensive gain of chr13 was significantly correlated with stage III and IV (Fisher exact test, p = 0.02). Interestingly, the gain in the chr31 was associated with a shorter duration of remission (117 days vs 300 days, p = 0.009).

Other 4 gains were found to be specific of therapy-resistant DLBCLs: CFA 3q34 (74.56 to 74.61 Mb), CFA 26q13 (12.68 to 12.69 Mb), CFA 23q11 (0.92 to 0.95 Mb) which includes TCEA3, and CFA 33q14 (17.27 to 17.29 Mb) that comprises CD200R1, a receptor restricted to the surface of myeloid lineage cells. Noteworthy, the segmentation algorithm employed by GISTIC showed a sub-regional variation along the CNA identified in chr8 (loss of Mb 72.95 to 74.12) by ADM-1 algorithm. By applying GISTIC approach, the 65% of this region, containing the canine IGH variable (IGHV) locus, was lost in both groups of dogs, while the incidence of the loss of the first 0.4 Mb, that holds four members of IGH constant gamma (IGHG1, IGHG2, IGHG3 and IGHG4), was significantly elevated in the therapy-resistant dogs.

### Comparative analysis of genomic imbalance in canine and human DLBCL

Orthologous chromosomal regions in canine and human genome were examined to assess evolutionary conserved CNAs between cDLBCL and human DLBCL (hDLBCL) (Table 2). To achieve this comparison, data of several studies describing frequent chromosome imbalances in activated B-cell-like (ABC) and germinal center B-cell-like (GCB) DLBCLs were employed [5], [15]–[19].

**Table 2:** Orthologous chromosomal regions in canine and human DLBCL.

hDLBCL					
CFA location	CNA	HSA location	CNA	Ref	Genes
13q21.1-q22	Gain	1p36.11	Gain	[19]	RUNX3
13q21.1-q22	Gain	7p13	Gain	[15], [16]	DBNL, PGAM2
13q21.1-q22	Gain	8q24	Gain	[15], [16]	MYC, TMEM75, NDRGI, HHLA1, ASAP1, PTK2
13q12-q13	Gain	18q21.1	Gain	[15]–[18]	RPL17, SMAD4
13q12-q13	Gain	16p12	Gain	[15], [16]	SYT17
13q21.1-q22	Gain	19q13	Gain	[15]–[18]	QPCTL, SNRPD2, FTL
13q21.1-q22	Gain	4q12	Loss	[18]	KIT, PDGFRα
17q15	Loss	2p11	Loss	[15], [16]	IGK locus
8q33	Loss	1q44	Gain	[15]	NLRP3

Comparative analysis of CNAs highlighted several regions shared between the two species. Within the gains, Homo Sapiens (HSA) 1p36.11, 7p13, 8q21-q26, 16p11-p13, 18q21.1 have been described to be frequently associated with pathogenesis of hDLBCL, and in particular HSA 8q24 and 19q13 are known to be responsible for amplification of MYC and TGFB1, respectively. Similar imbalances were found in the dog, and related to the extensive gain of canine chr13. Of interest are loss of HSA 4p12 and 4q12 in hDLBCL [18], which comprise KIT and PDGFRα. The canine orthologous region (CFA 13q21.1-q22) resulted significantly imbalanced in cDLBCL, although a gain, instead of loss, was observed in 58% of dogs.

An accurate analysis comparing imbalances at the IG loci between canine and human DLBCL revealed a frequent deletion encompassing the canine IGL locus (CFA 26q22) (91% cases), that has not been described in hDLBCL so far. On the other hand, the high resolution provided by the Agilent CGH platform allowed to identify two additional losses in the canine IGH locus (CFA 8q33) and IGK (CFA 17q16), in 91% and 100%, respectively. The latter corresponds to the loss in human chr2 (HSA 2p11) with a high frequency of IGK deletions, as reported in both ABC and GCB-hDLBCLs.

### Pathway Analysis

The DAVID analysis of recurrent CNAs in cDLBCLs was performed first by considering regions of gain and loss separately, and then by considering all aberrant intervals together. Functional annotations of gained regions identified a total of 52 enriched BP terms, the majority of which (16 out of 52) were related to Nucleotide biosynthesis (e.g. GO:0009167∼purine ribonucleoside monophosphate metabolic process, GO:0009123∼nucleoside monophosphate metabolic process). The three most enriched terms (>10-fold enriched) were all related to Inosine Monophosphate (IMP) metabolic process. The most significantly enriched KEGG pathway term was Ascorbate and Aldarate metabolism (hsa00053, p<0.001 Fold-enrichment >10). When considering only intervals of losses, 8 BP terms were found significantly enriched. The most significant one was Immune response (GO:0006955, p<0.01), which was represented mainly by IG locus. The remaining 7 BP terms were all related to protein synthesis (i.e. GO:0000028∼ribosomal small subunit assembly) and transcription initiation (GO:0006367∼transcription initiation from RNA polymerase II promoter, GO:0006357∼regulation of transcription from RNA polymerase II promoter). The only KEGG pathway significantly enriched in loss regions was Huntington’s disease (hsa05016). Functional annotation of all CNAs without distinguishing between gains and losses identified 55 enriched BP terms, mostly overlapping (42 out of 55) with those found to be enriched in gain intervals. Similar results were obtained when looking for enriched KEGG pathways, where, in addition to Ascorbate and aldarate metabolism and Huntington’s disease pathways, steroid hormone metabolism pathways (i.e. hsa00150: Androgen and oestrogen metabolism and hsa00140: Steroid hormone biosynthesis, p<0.001) were the most significant.

## Discussion

Genetic instability, clonal selection and evolution are important driving forces of cancer progression, and the resulting genetic heterogeneity is a hallmark of different clinical behavior. Oligo aCGH has been applied to several human tumors, revealing a high degree of heterogeneity within similar histotypes, and recurrent regions of copy-number abnormalities in the tumoral genomic DNA led to the discovery of genes that drive disease pathogenesis [7]–[9], [11].

When specifically considering hDLBCL, aCGH identified chromosomal aberrations that were significantly more frequent in a particular DLBCL subtype than in the others, thereby helping re-classifying DLBCLs and identifying distinct disease entities that are associated with clinical outcome [5].

Canine DLBCL is highly chemo-sensitive, and dose-intense multidrug chemotherapy may lead to prolonged survival [20]; however, some DLBCLs do not respond to therapy. Identifying CNAs associated with response to chemotherapy or survival could be beneficial to dogs with a poor prognosis, for which new treatments may be sought. In the present study, oligo aCGH was applied in dogs with DLBCL, by pairing tumoral DNA with normal DNA obtained through skin biopsies. DNA obtained from control tissues within the same subject allowed to find somato-genetic aberrations related to DLBCL, thereby excluding possible polymorphic genomic variations correlated with breed. Overall, aCGH provided a comprehensive high-resolution scanning of the DLBCL genome and identified multiple regions of recurrent copy number changes, one of which was significantly correlated with a shorter duration of remission.

The analysis of the genes located within the chromosomal segments with recurrent alterations in at least two dogs identified a total of 1,363 genes in pre-treatment DLBCLs. A number of known and previously unknown oncogenes were identified, some of which have already been reported to be associated with cancer, including: ADCY8, ZFAT, BAI1, PTK2, FBXL6, FOXH1, HSF1, and UGT2A1. The DAVID analysis, considering genes belonging to gain regions, highlighted numerous molecular pathways significantly enriched. This was mainly due to the recurrent gain of two whole chromosomes, chr13 and chr31 that, even if being attributable to “single events”, contributed with an extremely high number of genes. Interestingly, the great majority of enriched BP terms was related to nucleotide biosynthesis and IMP metabolism, rate-limiting step for purine synthesis, and therefore playing an important role in the regulation of cell growth and malignancy development. This finding was confirmed also by KEGG analysis, with the most enriched term being Ascorbate and aldarate metabolism, pathway reported to be dis-regulated in breast and ovarian cancer in humans and linked to nucleotide metabolism. The only KEGG term enriched in loss regions was Huntington’s disease, represented mainly by genes involved in oxydative phosphorylation (OxPhos). This finding is of particular interest if considering that OxPhos deficits have been recently associated with malignancies and tumor growth [21], [22]. When considering only deleted regions, the most significant BP pathway was the Immune response. This was not surprising, given the extremely high frequency of IG heavy and light chain loci deletions detected in cDLBCL that involve both IGK, IGL and IGH. Even if the canine IGH locus is minimally annotated, comparison with human orthologous genes locates the canine IGH locus in CFA 8q33 (72.95 to 74.12 Mb), a region frequently found to be deleted in cDLBCL (91%). The loss of IGK (CFA 17q15) in pre-treatment DLBCLs was found in all cases (100%), and the same region was also identified in the three relapsed DLBCLs and in the lymph nodes of two dogs that were in remission at the end of therapy. Similar evidence was found for the IGL locus (CFA 26q22), deleted in 91% of pre-treatment DLBCLs and in the three relapsed dogs.

A previous study with BACs in dogs with B-cell lymphoma reported a similar frequency for the IGL deletion, but the loss of IGK was less common [6]. One possible explanation for this discordance is the different technical approach. High-density oligo arrays are able to identify microaberrations that are beyond the limits of detection of the BAC array platform. In addition, different B-cell lymphoma histotypes were included in the previous study. Based on our results, clonal rearrangement of immunoglobulin light and heavy chains is manifested as a recurrent loss of IGK, IGL and IGH, thereby becoming a hallmark event in DLBCL. These new findings are in concordance with those referring to human DLBCL [23].

As previously mentioned, the loss of IGK locus is a common event also in human B-cell lymphomas (BCL) [16], while no chromosomal deletions have been reported for IGL (HAS 22q11) or IGHV (HSA 14q32) genes, even if these loci are common targets for rearrangements in hBCL. Indeed, a hallmark of hBCLs are balanced chromosomal translocations involving the IGHV locus. The most frequent are t(14; 18) (q32; q21) and t(8; 14) (q24; q32), that give rise to the BCL-2/Ig and MYC/Ig fusions, respectively [24]. These translocations, besides resulting in dysregulated expression of oncogenes, cause disruption and non-functionality of the involved IGH chain gene [25], [26]. A recent study [27] also demonstrated that MYC translocation with IG partner gene has a negative prognostic impact compared with non IG-MYC translocation. IGHV, IGK variable (IGKV) and IGL variable (IGLV) genes inactivation through destructive mutations is a well-known event also in post-transplant lymphoproliferative disorders (PTLDs), in particular in those originating from Germinal Center B cells [28]. Taken together, these evidences clearly demonstrate that in both dogs and humans IG heavy and light chain loci are commonly inactivated, possibly playing an essential role in DLBCL pathogenesis.

Remarkable in this context are the preliminary results of the GISTIC analysis, which identified additional losses of the IGHG locus as significantly associated with chemo-resistant cDLBCLs. Although further investigations are required, this finding suggests that the magnitude of IG loci losses may have clinical/prognostic relevance for cDLBCL.

In newly diagnosed DLBCLs, gains along the length of the chr13 were often observed in different regions. Interestingly, the segments where MYC (∼25.2 Mb), LDHB (∼28.3 Mb), PDGFRα (∼47.7 Mb) and KIT (∼47.1 Mb) are encoded were the most frequently involved (66–75%). Deregulation of MYC has been described to have a central role in cellular processes such as proliferation, differentiation, and metabolism. Aberrations involving MYC, in terms of both gains of 8q24 or translocations t(8; 14) (q24; q32), are a common event of hBCL and are associated with a more aggressive phenotype and poor outcome in human DLBCL, including shorter progression-free interval and overall survival [29]. In the present study, the gain of the whole canine chr13 was observed more frequently in therapy-responsive DLBCLs (5/6, compared to 2/6 therapy-resistant cDLBCLs); however, when looking specifically at the region containing MYC (24.3 to 26.3 Mb), the number of therapy-resistant DLBCLs showing imbalance doubled in size. This finding clearly supports the involvement of MYC in the pathogenesis of canine lymphoma, although further studies in a higher number of cases are needed to verify its association with clinical behavior in cDLBCL. LDHB is a critical enzymatic activator of glycolysis and the co-respective iso-enzymes are formed by the random combination of two different subunits encoded by two structurally distinct genes, LDHA and LDHB. Expression of mammalian LDHA and LDHB is regulated during development and is tissue specific; therefore, alterations in the LDH levels serve as indicators of pathologic involvement and cancer development [30]. The significance and the regulation of LDHB expression in cancer development remains unclear, but different tumor phenotypes may originate from the alteration of LDHA and LDHB caused by mutation, chromosomal deletion/duplication, and increase of copy number [31]. LDH levels at the end of chemotherapy have been also considered prognostic in canine lymphoma by predicting early recurrence [32].

PDGFRα is a type III receptor tyrosine kinase and genetic mutations have been reported in many human cancers [33]–[35]. In humans, these receptors have drawn much attention because they can be efficiently targeted by tyrosine kinase inhibitors. In this study, gain of PDGFRα was found in eight pre-treatment DLBCLs (67%) 3 of which relapsed. The dogs in clinical remission at the end of chemotherapy never showed CNAs in this region. The gain of PDGFRα was confirmed in a previous study [36] where PDGFRα expression was found increased in cDLBCLs compared to healthy controls.

It is worth noticing that although PDGFRα and KIT are expressed in a variety of human hematopoietic neoplasms, the chromosomal region where both genes are located (HSA 4q12) is frequently deleted in hDLBCL [18], evidence totally in contrast with what observed herein.

In the present study, for some dogs aCGH analysis was repeated at the end of treatment or at relapse. When we reconsidered all CNAs based on the clinical follow-up, we found 701 genes out of 1,363 showing recurrent mutations exclusively in dogs that did not relapse during treatment. These genes are mainly related to the gain of canine chr13 that we found to be significantly more frequent in therapy-responsive DLBCLs.

A total of 656 genes were shared between not relapsing and relapsing dogs while, notably, imbalances of 6 genes (PTGES3L, AOC3, AOC2, MPP6, BMP2 K and PAQR3) were found exclusively in dogs that relapsed before the end of treatment. Interestingly, PAQR3 deletion is known to enhance tumorigenesis in mouse models, whereas the reduced expression in human colorectal cancer is associated with the regulation of the proliferation [37]. Its role in lymphoma is unknown, but this data opens new perspectives in this tumor.

In relapsed DLBCLs, a reduced number of chromosomal rearrangements was found when compared with the corresponding newly-diagnosed DLBCLs. One dog had the same profile, suggesting a linear clonal evolution; however, the other two dogs were more complicated in terms of aberrations, and new rearrangements were detected. Two different scenarios can explain these results. First, chemotherapy alters the growth conditions of the neoplastic cells, thereby having a strong impact on the selection process of the resistant clones. If neoplastic cells dominating at diagnosis are sensitive to therapy, minor clones with intrinsic resistance may be secondarily generated by the tumor, and expand more efficiently once the competing clones are eliminated by the treatment. In the second hypothesis, these clones were present but undetectable in the pre-treatment lymphoma and became evident at relapse [38]. In the dog with the same pattern of chromosomal rearrangements, chemotherapy seemed ineffective for the neoplastic clones. Future studies should be directed on the impact of cell growth properties and the clonal selection process in canine DLBCL before and after treatment.

Interestingly, in dogs that were in clinical and molecular remission at the end of chemotherapy, an unexpected number of new gains and losses were found. A possible explanation of this finding might be related to the fact that a reduced number of clonal cells are still present in the lymph node, but the number of chromosomal arrangements is insufficient for lymphoma identification both by PARR or clinically. The histology of the lymph nodes was similar, being characterized by atrophic follicles. The paracortex displayed a moderate proliferation of the high endothelial venules and a high number of plasma cells were also present. These features were attributed to the effects of chemotherapy. Surprisingly, 3 dogs in remission at the end of treatment exhibited a new loss in CFA 8q11 (2.4 to 2.9), where T-cell receptor (TCR) alpha and delta genes are located. Moreover, one of them showed a loss in chr16, where TCR beta is located. These results are analogous to the findings observed in human melanoma after administration of a vaccine consisting of autologous melanoma cells modified with dinitrophenyl [39]. In these patients, a TCR rearrangement in lymphocytes within the lesion was found. These three dogs received an autologous therapeutic vaccine in addition to chemotherapy, as previously described. The vaccine consisted of hydroxyapatite, heat shock proteins and proteins from the cell membrane system. Likewise human melanoma, immunotherapy might induce TCRs rearrangements elicited by lymphoma-associated antigens, thereby explaining the results obtained here.

In conclusion, the current results demonstrate that the application of oligo aCGH contributed to identifying CNAs that provided new insight for the study of cDLBCL. Preliminary statistical analysis highlighted regions associated with a poor outcome in a group of dogs. Thus, our analysis provides a rich starting point for future investigations into the molecular pathogenesis of canine DLBCL.

## Supporting Information

File S1 **Supporting tables. Table S1,** Selected dogs and main clinical data. **Table S2,** List of CNAs in pre-treatment DLBCLs. **Table S3,** List of CNAs in selected post-treatment dogs.
(DOCX)
