# Supplementary material for: Array-Based Comparative Genomic Hybridization Analysis Reveals Chromosomal Copy Number Aberrations Associated with Clinical Outcome in Canine Diffuse Large B-Cell Lymphoma
Source: PLoS One. 2014 Nov 5;9(11):e111817. doi: 10.1371/journal.pone.0111817 (PMC4221131; doi:10.1371/journal.pone.0111817)
Supplement: File S1 — Supporting tables. Table S1, Selected dogs and main clinical data. Table S2, List of CNAs in pre-treatment DLBCLs. Table S3, List of CNAs in selected post-treatment dogs. (DOCX) [file pone.0111817.s001.docx]

**Table S1.** Selected dogs and main clinical data

| **Cases** | **Chemotherapy** | **Chemo-Immunotherapy** | **Response to therapy** | **Diagnosis end-staging** |
| --- | --- | --- | --- | --- |
| 1 * |  | X | responsive | reactive hyperplasia |
| 2 * |  | X | responsive | reactive hyperplasia |
| 3 |  | X | responsive | reactive hyperplasia |
| 4 |  | X | responsive | reactive hyperplasia |
| 5 | X |  | resistant | DLBCL |
| 6 | X |  | resistant |  |
| 7 |  | X | resistant |  |
| 8 | X |  | resistant |  |
| 9 |  | X | responsive |  |
| 10 |  | X | resistant |  |
| 11 |  | X | responsive |  |
| 12 |  | X | resistant |  |

* cases relapsed after the end of therapy

**Table S2.** List of CNAs in pre-treatment DLBCLs

| **n° aberration** | **Chr** | **Cytogenetic band** | **Start position (Mb)** | **Stop position (Mb)** | **Type of aberration** | **Genes** | **Frequency (%)** |
| --- | --- | --- | --- | --- | --- | --- | --- |
| 1 | 1 | 1q14 | 38.25 | 38.29 | Gain | 1 | 8% |
| 2 | 1 | 1q22 | 49.19 | 49.24 | Gain | 1 | 8% |
| 3 | 1 | 1q38 | 121.10 | 121.13 | Loss | 1 | 16% |
| 4 | 1 | 1q31-q38 | 95.28 | 122.32 | Loss | 821 | 8% |
| 5 | 2 | / | 53.45 | 53.65 | Gain | 1 | 8% |
| 6 | 3 | 3q12.1-q12.4 | 10.52 | 19.07 | Loss | 29 | 8% |
| 7 | 3 | 3q12.4 | 19.08 | 21.49 | Loss | 4 | 16% |
| 8 | 3 | 3q21.1 | 21.50 | 22.99 | Loss | / | 8% |
| 9 | 3 | 3q22.1 | 38.98 | 39.01 | Gain | 1 | 8% |
| 10 | 3 | 3q34 | 74.57 | 74.61 | Gain | 1 | 8% |
| 11 | 5 | 5q14.3 | 19.35 | 27.14 | Gain | 63 | 8% |
| 12 | 6 | 6q11 | 33.67 | 33.71 | Gain | / | 8% |
| 13 | 6 | 6q11 | 37.62 | 41.45 | Loss | 171 | 8% |
| 14 | 6 | 6q22 | 43.95 | 44.43 | Gain | 2 | 8% |
| 15 | 6 | 6q24.1-q25.1 | 64.33 | 74.60 | Loss | 32 | 8% |
| 16 | 7 | 7q21.1 | 50.53 | 50.56 | Gain | / | 8% |
| 17 | 8 | 8q13 | 14.49 | 14.51 | Gain | 1 | 8% |
| 18 | 8 | / | 29.08 | 29.11 | Gain | 1 | 8% |
| 19 | 8 | 8q33.1 | 62.89 | 62.92 | Gain | 1 | 8% |
| 20 | 8 | 8q33.2 | 70.40 | 70.86 | Loss | 7 | 8% |
| 21 | 8 | 8q33.2-q33.3 | 71.45 | 72.87 | Loss | 34 | 8% |
| 22 | 8 | 8q33.3 | 72.95 | 74.12 | Loss | 13 | 91% |
| 23 | 9 | 9q11.1 | 0.02 | 5.44 | Loss | 142 | 8% |
| 24 | 9 | 9q11.1 | 0.91 | 1.00 | Loss | 3 | 16% |
| 25 | 9 | 9q11.1 | 1.01 | 1.03 | Loss | 1 | 16% |
| 26 | 9 | 9q14 | 20.17 | 20.19 | Gain | 3 | 16% |
| 27 | 10 | 10q14-q21 | 15.92 | 24.37 | Gain | 117 | 16% |
| 28 | 10 | 10q21 | 54.69 | 54.73 | Gain | 3 | 16% |
| 29 | 10 | 10q11-q27.2 | 0.06 | 69.25 | Gain | 618 | 8% |
| 30 | 11 | 11q12.3 | 18.17 | 18.34 | Gain | 1 | 8% |
| 31 | 11 | 11q16-q21 | 50.52 | 53.03 | Loss | 65 | 8% |
| 32 | 11 | 11q22 | 68.80 | 68.88 | Gain | / | 8% |
| 33 | 12 | / | 24.53 | 24.56 | Gain | 1 | 8% |
| 34 | 12 | 12q23 | 59.31 | 61.21 | Loss | 2 | 8% |
| 35 | 13 | 13q11-q12 | 0.03 | 16.67 | Gain | 64 | 50% |
| 36 | 13 | 13q12-q13 | 16.68 | 24.48 | Gain | 44 | 50% |
| 37 | 13 | 13q13 | 24.35 | 26.26 | Gain | 1 | 75% |
| 38 | 13 | 13q13-q21.2 | 26.28 | 34.46 | Gain | 23 | 66% |
| 39 | 13 | 13q21.1 | 34.47 | 37.43 | Gain | 46 | 58% |
| 40 | 13 | 13q21.1 | 37.44 | 38.22 | Gain | 59 | 66% |
| 41 | 13 | 13q21.1-22.2 | 38.22 | 63.08 | Gain | 145 | 58% |
| 42 | 14 | 14q11.2 | 38.19 | 38.20 | Loss | 1 | 16% |
| 43 | 14 | 14q11.2 | 40.33 | 40.42 | Loss | 5 | 16% |
| 44 | 14 | 14q11.2 | 40.80 | 41.01 | Loss | 2 | 16% |
| 45 | 14 | 14q21 | 57.92 | 58.21 | Loss | 2 | 8% |
| 46 | 14 | Whole | 0.47 | 60.93 | Loss | 362 | 8% |
| 47 | 15 | 15q13 | 14.55 | 14.96 | Loss | 11 | 8% |
| 48 | 15 | 15q13-q21 | 15.69 | 17.52 | Loss | 30 | 8% |
| 49 | 15 | 15q24.3 | 53.51 | 54.06 | Loss | 1 | 8% |
| 50 | 16 | 16q12 | 9.49 | 9.74 | Gain | 5 | 16% |
| 51 | 16 | 16q13 | 14.64 | 15.21 | Gain | 26 | 16% |
| 52 | 16 | 16q21 | 29.35 | 29.38 | Gain | 1 | 16% |
| 53 | 16 | 16q21 | 33.55 | 33.77 | Gain | 2 | 16% |
| 54 | 16 | 16q11-q25.2 | 0.40 | 59.52 | Gain | 478 | 8% |
| 55 | 17 | 17q11 | 0.22 | 1.28 | Gain | 7 | 16% |
| 56 | 17 | 17q15 | 37.53 | 37.60 | Loss | 1 | 50% |
| 57 | 17 | 17q15 | 37.69 | 37.85 | Loss | 1 | 100% |
| 58 | 17 | 17q21 | 50.31 | 50.36 | Gain | 1 | 16% |
| 59 | 17 | 17q11-q24 | 0.22 | 64.18 | Gain | 587 | 8% |
| 60 | 18 | 18q22.1-q22.2 | 29.13 | 30.74 | Loss | 1 | 8% |
| 61 | 19 | 19q21? | 20.03 | 20.32 | Loss | 1 | 16% |
| 62 | 20 | 20q17 | 57.05 | 58.02 | Loss | 54 | 16% |
| 63 | 21 | 21q11-q24.3 | 0.41 | 50.73 | Gain | 454 | 8% |
| 64 | 22 | 22q11-q11.2 | 0.51 | 6.48 | Loss | 46 | 8% |
| 65 | 23 | 23q11 | 0.92 | 0.99 | Gain | / | 8% |
| 66 | 23 | 23q22 | 34.89 | 35.12 | Loss | 1 | 8% |
| 67 | 24 | 24q24-q25 | 41.99 | 47.61 | Loss | 86 | 8% |
| 68 | 25 | 25q12 | 9.35 | 10.92 | Gain | 7 | 8% |
| 69 | 25 | 25q24 | 48.69 | 51.61 | Loss | 42 | 8% |
| 70 | 25 | 25q24 | 50.32 | 50.50 | Loss | 1 | 41% |
| 71 | 26 | 26q22 | 25.41 | 27.63 | Loss | 21 | 91% |
| 72 | 27 | 27q11 | 1.04 | 1.18 | Loss | 2 | 8% |
| 73 | 27 | 27q22.1-q22.2 | 36.63 | 37.67 | Loss | 20 | 8% |
| 74 | 27 | 28q11-q18 | 0.17 | 45.77 | Gain | 480 | 16% |
| 75 | 28 | 28q18 | 40.92 | 41.13 | Loss | / | 16% |
| 76 | 28 | 28q18 | 39.68 | 40.40 | Loss | / | 16% |
| 77 | 28 | 28q17-q18 | 38.45 | 41.08 | Loss | 24 | 8% |
| 78 | 29 | 29q12 | 7.55 | 7.59 | Gain | 1 | 25% |
| 79 | 29 | 29q21 | 23.70 | 25.59 | Loss | 3 | 8% |
| 80 | 29 | 29q11-q23.2 | 0.07 | 41.74 | Gain | 174 | 8% |
| 81 | 30 | 30q14.3 | 23.37 | 23.42 | Gain | / | 8% |
| 82 | 31 | 31q12-q15.2 | 12.00 | 35.91 | Gain | 122 | 50% |
| 83 | 31 | 31q15.2-q15.3 | 36.26 | 39.82 | Loss | 67 | 8% |
| 84 | 31 | Whole | 0.09 | 39.82 | Gain | 202 | 41% |
| 85 | 32 | 32q11.1 | 3.27 | 3.30 | Gain | 2 | 25% |
| 86 | 32 | 32q11.2-q15.2 | 0.22 | 38.54 | Gain | 191 | 8% |
| 87 | 33 | 33q15.1 | 20.94 | 21.82 | Loss | / | 8% |
| 88 | 34 | 34q17 | 40.59 | 40.68 | Loss | 1 | 8% |
| 89 | 36 | 36q11 | 4.16 | 4.23 | Gain | 1 | 8% |
| 90 | 38 | 38p15.2 | 23.34 | 23.76 | Loss | 6 | 8% |

**Table S3.** List of CNAs in selected post-treatment dogs

|  |  |  |  |  | **Cases** | | | | | | | | | | | |
| --- | --- | --- | --- | --- | --- | --- | --- | --- | --- | --- | --- | --- | --- | --- | --- | --- |
| **Chr** | **Cytogenetic band** | **Start position (Mb)** | **Stop position (Mb)** | **Type of aberration** | **1** | **1 #** | **1 ##** | **2** | **2 #** | **2 ##** | **3** | **3 #** | **4** | **4 #** | **5** | **5 ##** |
| 1 | 1q38 | 121.10 | 121.13 | Loss | X |  |  |  |  |  |  |  |  |  |  |  |
| 3 | 3q12.4 | 19.08 | 22.99 | Loss |  |  |  |  |  |  | X |  |  |  |  |  |
| 6 | 6q24.1-q25.1 | 64.33 | 74.60 | Loss | X |  |  |  |  |  |  |  |  |  |  |  |
| 8 | 8q11 | 2.42 | 2.91 | Loss |  |  |  |  | X | X |  | X |  | X |  |  |
| 8 | 8q33.2 | 70.71 | 70.76 | Loss |  |  |  |  |  | X |  |  |  |  |  |  |
| 8 | 8q33.3 | 72.95 | 74.12 | Loss | X |  | X | X |  | X | X | X | X | X |  |  |
| 9 | 9q11 | 0.91 | 1.00 | Loss | X |  |  |  |  |  |  |  |  |  |  |  |
| 9 | 9q11 | 1.01 | 1.03 | Loss | X |  |  |  | X |  |  |  |  |  |  |  |
| 10 | 10q11-q27 | 0.06 | 69.25 | Gain |  |  |  |  |  |  | X |  |  |  |  |  |
| 12 | 12q23 | 59.31 | 61.21 | Loss | X |  |  |  |  |  |  |  |  |  |  |  |
| 13 | 13q11-q12.1 | 0.03 | 16.67 | Gain | X |  | X | X |  | X | X |  |  |  |  |  |
| 13 | 13q12.1-q13 | 16.68 | 24.48 | Gain | X |  | X | X |  | X |  |  |  |  |  |  |
| 13 | 13q13 | 24.35 | 26.26 | Gain | X |  | X | X |  | X | X |  |  |  | X | X |
| 13 | 13q13-q21.1 | 26.28 | 34.46 | Gain | X |  | X | X |  | X | X |  |  |  | X | X |
| 13 | 13q21.1 | 34.47 | 37.43 | Gain | X |  | X | X |  | X | X |  |  |  | X | X |
| 13 | 13q21.1 | 37.44 | 38.22 | Gain | X |  | X | X |  | X | X |  |  |  | X | X |
| 13 | 13q21.1-q22.2 | 38.22 | 63.08 | Gain | X |  | X | X |  | X | X |  |  |  | X | X |
| 14 | 14q23 | 57.92 | 58.21 | Loss | X | X | X |  |  |  |  |  |  |  |  |  |
| 15 | 15q13-q21 | 15.69 | 17.52 | Loss |  |  |  |  |  |  | X |  |  |  |  |  |
| 15 | 15q24.3-q25 | 53.51 | 54.06 | Loss | X |  |  |  |  |  |  |  |  |  |  |  |
| 16 | 16q11 | 1.96 | 1.99 | Loss |  |  |  |  |  | X |  |  |  |  |  |  |
| 16 | 16q12 | 6.77 | 6.96 | Loss |  |  |  |  | X |  |  |  |  |  |  |  |
| 16 | 16q13 | 14.64 | 15.21 | Loss |  |  |  |  |  |  | X |  |  |  |  |  |
| 16 | 16q11-q25.2 | 0.40 | 59.52 | Gain |  |  |  |  |  |  |  |  | X |  |  |  |
| 17 | 17q15 | 37.53 | 37.60 | Loss | X |  |  |  |  |  | X |  | X |  |  |  |
| 17 | 17q15 | 37.69 | 37.85 | Loss | X | X | X | X |  | X | X |  | X | X | X | X |
| 17 | 17q21 | 50.31 | 50.36 | Loss |  |  |  |  |  |  |  |  | X |  |  |  |
| 18 | 18q22.1-q22.2 | 29.13 | 30.74 | Loss | X |  |  |  |  |  |  |  |  |  |  |  |
| 19 | 19q21 | 20.29 | 20.32 | Loss |  | X |  |  |  |  |  |  |  |  |  |  |
| 20 | 20q17 | 57.05 | 58.02 | Loss |  |  |  |  |  |  | X |  |  |  |  |  |
| 25 | 25q24 | 50.32 | 50.50 | Loss | X |  |  |  |  |  | X |  | X |  |  |  |
| 25 | 25q24 | 51.16 | 51.22 | Gain |  |  |  |  | X |  |  |  |  |  |  |  |
| 26 | 26q22 | 25.41 | 27.63 | Loss | X | X | X | X |  | X | X | X | X | X | X | X |
| 27 | 27q11-q22.3 | 0.17 | 45.77 | Gain |  |  |  |  |  |  | X |  | X |  |  |  |
| 28 | 28q18 | 39.68 | 40.40 | Loss |  |  |  |  |  |  | X |  |  |  |  |  |
| 28 | 28q18 | 40.92 | 41.13 | Gain | X |  |  |  |  |  |  |  |  |  |  |  |
| 29 | 29q21 | 23.70 | 25.59 | Loss | X |  |  |  |  |  |  |  |  |  |  |  |
| 31 | 31q11-q15.3 | 0.09 | 39.82 | Gain |  |  |  | X |  | X |  |  |  |  |  |  |
| 33 | 33q15.1 | 20.94 | 21.82 | Loss |  |  |  |  |  |  |  |  | X |  |  |  |
| 34 | 34q17 | 40.59 | 40.68 | Loss |  |  |  |  |  |  |  |  | X |  |  |  |
| 37 | 37q14 | 18.93 | 18.96 | Loss |  |  |  |  |  |  |  | X |  |  |  |  |
| 38 | 38q15.2 | 23.34 | 23.76 | Loss |  |  |  | X |  |  |  |  |  |  |  |  |

# = dogs in clinical remission

## = relapsed DLBCLs
